# Stability of OCT and OCTA in the Intensive Therapy Unit Setting

**DOI:** 10.3390/diagnostics11081516

**Published:** 2021-08-23

**Authors:** Ella F. Courtie, Aditya U. Kale, Benjamin T. K. Hui, Xiaoxuan Liu, Nicholas I. Capewell, Jonathan R. B. Bishop, Tony Whitehouse, Tonny Veenith, Ann Logan, Alastair K. Denniston, Richard J. Blanch

**Affiliations:** 1 Neuroscience and Ophthalmology, Institute of Inflammation and Ageing, College of Medical and Dental Sciences, University of Birmingham, Birmingham B15 2TT, UK; efc526@student.bham.ac.uk; 2 Ophthalmology Department, University Hospitals Birmingham NHS Foundation Trust, Birmingham B15 2TH, UK; a.kale@nhs.net (A.U.K.); b.hui@nhs.net (B.T.K.H.); xiaoxuan.liu@nhs.net (X.L.); nicholas.capewell@uhb.nhs.uk (N.I.C.); a.denniston@bham.ac.uk (A.K.D.); 3 NIHR Surgical Reconstruction and Microbiology Research Centre, University Hospitals Birmingham NHS Foundation Trust, Birmingham B15 2TH, UK; j.bishop.1@bham.ac.uk; 4 Academic Unit of Ophthalmology, Institute of Inflammation and Ageing, College of Medical and Dental Sciences, University of Birmingham, Birmingham B15 2TT, UK; 5 Health Data Research UK, London NW1 2BE, UK; 6 Moorfields Eye Hospital NHS Foundation Trust, London EC1V 2PD, UK; 7 Critical Care Unit, University Hospitals Birmingham NHS Foundation Trust, Birmingham B15 2TH, UK; tony.whitehouse@uhb.nhs.uk (T.W.); tonny.veenith@uhb.nhs.uk (T.V.); 8 Birmingham Acute Care Research Group, Institute of Inflammation and Ageing, College of Medical and Dental Sciences, University of Birmingham, Birmingham B15 2TT, UK; 9 Department of Trauma Sciences, University of Birmingham, Birmingham B15 2TT, UK; 10 Axolotl Consulting Ltd., Worcestershire, Droitwich WR9 0JS, UK; 11 Division of Biomedical Sciences, Warwick Medical School, University of Warwick, Coventry CV4 7HL, UK; ann.logan@warwick.ac.uk; 12 NIHR Biomedical Research Centre for Ophthalmology, Moorfields Eye Hospital NHS Foundation Trust and UCL Institute of Ophthalmology, London EC1V 2PD, UK; 13 Centre for Regulatory Science and Innovation, Birmingham Health Partners, Birmingham B15 2TT, UK; 14 Royal Centre for Defence Medicine, Academic Department of Military Surgery and Trauma, Birmingham B15 2TH, UK

**Keywords:** optical coherence tomography angiography, stability, critical care

## Abstract

To assess the stability of retinal structure and blood flow measures over time and in different clinical settings using portable optical coherence tomography angiography (OCTA) as a potential biomarker of central perfusion in critical illness, 18 oesophagectomy patients completed retinal structure and blood flow measurements by portable OCT and OCTA in the eye clinic and intensive therapy unit (ITU) across three timepoints: (1) pre-operation in a clinic setting; (2) 24–48 h post-operation during ITU admission; and (3) seven days post-operation, if the patient was still admitted. Blood flow and macular structural measures were stable between the examination settings, with no consistent variation between pre- and post-operation scans, while retinal nerve fibre layer thickness increased in the post-operative scans (+2.31 µm, *p* = 0.001). Foveal avascular zone (FAZ) measurements were the most stable, with an intraclass correlation coefficient of up to 0.92 for right eye FAZ area. Blood flow and structural measures were lower in left eyes than right eyes. Retinal blood flow assessed in patients before and during an ITU stay using portable OCTA showed no systematic differences between the clinical settings. The stability of retinal blood flow measures suggests the potential for portable OCTA to provide clinically useful measures in ITU patients.

## 1. Introduction

Optical coherence tomography (OCT) is a non-invasive laser imaging modality generating high-resolution cross-sectional retinal images, including the macula and retinal nerve fibre layer (RNFL) [1]. Optical coherence tomography angiography (OCTA) images retinal and choroidal blood flow three-dimensionally by using moving red blood cells as the contrast medium, without the need for injectable contrast [2].

Critically ill patients with sepsis and systemic inflammatory response syndrome often have systemic microcirculatory defects, manifested as reduced functional capillary density, which reduces oxygen delivery and propagates organ dysfunction [3]. Retinal microcirculation is readily quantifiable and can be sequentially monitored non-invasively. Retinal neuronal and microvascular changes mirror systemic and cerebral pathology in health and disease, such as in Parkinson’s and Alzheimer’s Disease [3,4,5,6], and retinal and cerebral circulations share similar mechanisms of blood flow regulation [7], suggesting that retinal blood flow may serve as a surrogate for cerebral perfusion [3]. OCTA demonstrates retinal blood flow derangements in microvascular diseases, such as atrial fibrillation [8], acute coronary syndrome [9], systemic hypertensive crisis [10], inflammatory bowel disease [11], haemorrhagic shock [12,13], high-risk pregnancies [14], and preeclampsia [15]. 

As critical illness often requires treatment in the intensive therapy unit (ITU) [16], and intra-hospital transport of ITU patients is associated with morbidity [17], patient movement into clinic to allow OCT and OCTA images to be taken on a standard table-top device is usually not possible. New portable OCT systems allow assessment of patients in the ITU [18], but the effect of imaging in these more challenging clinical locations has not been reported. We therefore conducted a pilot study aiming to investigate the stability of OCT and OCTA assessment in the ITU setting in patients with planned non-neurological ITU stays following oesophagectomy, through comparison of measurements taken from pre- and post-operative retinal imaging, in order to assess the potential clinical utility of portable OCT and OCTA measures as a monitoring tool in the ITU environment. 

## 2. Methods

### 2.1. Study Design and Setting

This was a prospective observational cohort study evaluating the stability of retinal OCT and OCTA imaging in patients before and after scheduled major non-neurological surgery requiring planned ITU admission. Patients were recruited under two different studies approved by the NHS Research Ethics Service (Defining Outcome Measures in Ocular Inflammatory Disease: 14/EM/1163; Ophthalmic and Neurocognitive Assessment in the Management of Critically Ill Patients: 19/YH/0113), and studies were conducted between March 2018 and February 2020 in the Ophthalmology Department and Critical Care Unit at the Queen Elizabeth University Hospitals Birmingham NHS Foundation Trust. 

Inclusion criteria were patients over the age of 18 with planned oesophagectomy. Exclusion criteria were individuals with pre-existing retinal pathology, optic nerve pathology, or known neurological conditions, which were assessed using patient history and the review of case notes. Written informed consent was obtained from each individual before pre-operative (pre-op) imaging. Oesophagectomy patients were the chosen patient cohort because post-operative (post-op) care is routinely performed on the ITU and the surgery is elective, therefore allowing researchers to identify and recruit eligible participants and perform baseline imaging before their ITU stay.

### 2.2. Acquisition Devices

Scans on 12 patients were taken with the portable SPECTRALIS^®^ flex Heidelberg HRA + OCT module (Heidelberg Engineering, Heidelberg, Germany) for all time points. Scans on 6 patients were taken on the SPECTRALIS^®^ Heidelberg OCT2 table-top module (Heidelberg Engineering, Heidelberg, Germany) at baseline and post-op with the flex module. 

### 2.3. Scanning Protocol

A total of 18 patients with planned ITU admission were imaged, with both eyes assessed where possible. The scanning protocol included three scans: “Fast Macula” OCT (25 B-scans over an area of 5.7 mm^2^, at an automatic real time (ART) setting of 9 A-scans, averaged); RNFL OCT (100 ART); and OCTA of the macula (512 B-scans over an area of 2.8 mm^2^, with an ART setting of 5). 

Baseline pre-op scans (4–32 days pre-op), 24–48 h post-op scans during ITU admission, and 7 day post-op scans of patients still in an ITU or ward setting were acquired. Pupil dilation using tropicamide 0.5% eye drop solution (Minims, Bausch & Lomb, Surrey, UK) was achieved to improve scan quality, with surface lubricant hyaluronic acid (Hyloforte, Scope Ophthalmics Ltd., Crawley, UK) applied where necessary [18]. 

### 2.4. OCTA Analysis 

Partially completed OCTA scans or those with substantial motion artefacts were excluded from the study (1 OCTA scan out of 79). Substantial motion artefacts were deemed as those scans that had large areas of missing data, such as dark lines due to motion, i.e., blinking [19], or white lines due to eye movement. 

**Figure 1:**
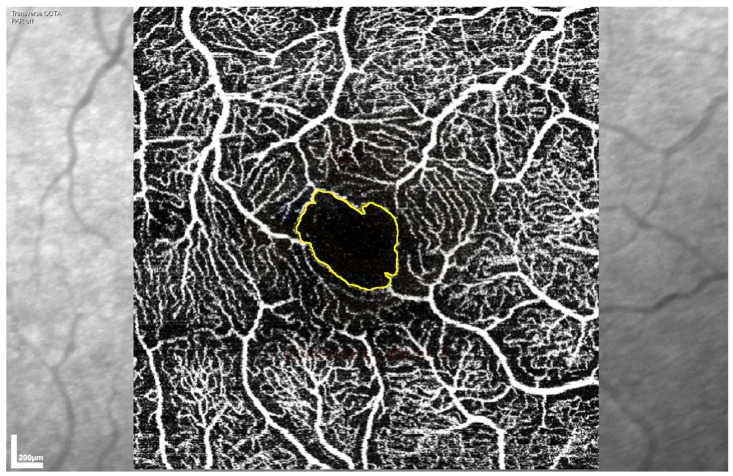
Representative low-power image of the foveal avascular zone (FAZ) area at the superficial vascular plexus, acquired by optical coherence tomography angiography (OCTA) and used to assess retinal perfusion. The FAZ is visible as the dark area in the centre of the scan with the drawn yellow outline.

Superficial vascular plexus (SVP) and intermediate capillary plexus (ICP) images were automatically segmented by the manufacturer’s software. Vascular morphology and vessel density metrics were calculated using a custom MatLab (MATLAB R2019a, MathWorks, Natick, MA) image processing algorithm to analyse both the SVP and ICP OCTA en-face images [20]. Vascular morphology was assessed by measuring skeletal fractal dimension (SFD), and vessel density assessed by skeletal vessel density (SVD), which are both expressed as arbitrary units. Foveal avascular zone (FAZ) area and FAZ perimeter were measured on both the SVP and ICP OCTA images, using the ImageJ Fiji program (National Institutes of Health, Bethesda MD, USA) [21]. The perimeter of the FAZ was marked manually (Figure 1) using the “Polygon” tool, with perimeter and area calculated within the program, adjusting the scale to ensure that perimeter was measured in mm and area in mm^2^ (“Scale” set to 303.7511 pixels/mm). 

### 2.5. RNFL and Macular Ganglion Cell Layer (GCL) Analysis

The manufacturer’s automated segmentation and analysis, with manual verification, was used to calculate RNFL and GCL thickness from vertical sections through the retina, with the RNFL thickness calculated in 7 segments (temporal superior, temporal, temporal inferior, nasal inferior, nasal, nasal superior, and global) and the macular GCL in 9 segments (outer superior, inner superior, outer nasal, inner nasal, outer inferior, inner inferior, outer temporal, inner temporal, and central). 

### 2.6. Statistics

Each scan for all eyes was analysed and compared, as were scans from both eyes of each patient, with the difference between patients also evaluated. OCT parameters compared between patients included RNFL and GCL thickness, with the OCTA parameters SFD, SVD, FAZ area and FAZ perimeter of the SVP and ICP also being considered. The sets of measurements for each segment were each analysed using a linear regression model adjusted for three fixed effects: scan number (1, 2, or 3), eye (left: OS; right: OD), and patient number. Results are summarised as mean differences with corresponding 95% confidence intervals and *p*-values. The level of agreement between the three scans in the sets of measurements for each segment is estimated using the intraclass correlation coefficient (ICC). The ICC values calculated for each set of measurements are ICC2 values based on the Shrout and Fleiss classification [22]. This assumes that a random sample of 3 scans have been performed on each patient, and the measure is one of absolute agreement in the measurements. Therefore, the ICC values calculated are based on two-way random effects, absolute agreement, and single rater/measurement models. ICC estimates for each combination of blood flow marker and eye were checked using linear mixed effects models, including scan and patient as random effects. The results were summarised as ICC values with 95% confidence intervals. All analyses were performed in R (v3.6.1) [23] and used the ggplot2 [24] and psych [25] packages.

## 3. Results

A total of 35 eyes from 18 patients were included in the final analysis. One patient had an unclear FAZ area in the ICP in all scans taken, and these figures were excluded from the final analysis. No patients were diagnosed with sepsis in the post-op period, and none required inotropic support, as detailed in Supplementary Table S1a,b.

### 3.1. Agreement of SVP and ICP Measures between Pre- and Post-Op Scans

Analysis of both SVP and ICP showed no evidence of a systematic difference in the mean SFD, SVD, FAZ area or FAZ perimeter between pre-op and the two post-op scans (Supplementary Figure S1, Table 1). ICC, as a measure of agreement for SVP and ICP, showed the highest agreement between ICP FAZ metrics (up to 0.92 for the right eye FAZ area; Table 2) and the worst for ICP SFD (0.06 for the right eye; Table 2). 

### 3.2. Agreement of RNFL, GCL, and Total Retinal Thickness between Pre- and Post-Op Scans

ICC varied from 0.55–0.94 for GCL thickness in individual retinal Early Treatment Diabetic Retinopathy Study (ETDRS) grid areas and 0.96–0.98 for global RNFL (Table 3). 

### 3.3. RNFL Thickness Increased Post-Op

There was strong evidence that global RNFL thickness was greater in the post-op scans than the pre-op scan, by 2.31 µm (*p* = 0.001). There was no change in macular GCL between pre-op and post-op scans.

### 3.4. ICP SFD and GCL Thickness Were Lower in Left than Right Eyes

Retinal blood flow assessed by SFD of ICP images was lower in left eyes than right eyes (Table 4; *p* = 0.05), with weak evidence that SVD of the ICP, and the SFD and SVD of the SVP, were lower in left than right eyes (*p* = 0.07, *p* = 0.08, and *p* = 0.14, respectively). FAZ perimeter was higher in left than right eyes in the ICP (Table 4; *p* = 0.01), with a non-significant trend that ICP FAZ area (*p* = 0.06) was also higher in the left eyes than right eyes (Table 4). However, there was no evidence that FAZ area and perimeter of the SVP were higher in left than right eyes (*p* = 0.40 and *p* = 0.62, respectively; Table 4). Taken together, these results suggest lower retinal blood flow in left compared to right eyes.

There was no difference in global RNFL thickness between left and right eyes, although some areas did show regional differences, with the temporal and temporal superior segments of left eyes being thinner than right eyes (Table 5; −2.737 µm, *p* = 0.005; −8.79 µm, *p* = 0.004, respectively). Multivariate analysis of macular GCL thickness in the ETDRS grid areas showed macular GCL thickness was lower in left than right eyes (average −5.37 µm, *p* = 2.764 × 10^−5^, Pillai test = 0.4633). 

## 4. Discussion

When performing structural OCT and OCTA on non-neurological critical care patients without haemodynamic instability, there was no systematic difference in blood flow measures or macular structural measures between pre-op images taken in a clinic setting and post-op images taken in the ITU. This supports the potential of OCTA to measure retinal blood flow in a critical care environment and suggests it could serve as central perfusion biomarkers in this patient cohort. A previous study by Liu et al. [18] showed the feasibility of using the portable SPECTRALIS flex module for taking OCT images in unconscious and critically ill patients in a critical care unit, also demonstrating the possibility of using this device in high-dependency areas.

FAZ measurements showed higher agreement between pre- and post-operative scans than SFD and SVD, with FAZ measurements showing the highest agreement in the ICP, while SFD and SVD showed the greatest agreement in the SVP. The SVP is the most reliable anatomical layer to assess SFD and SVD, as deeper retinal areas can experience projection artefacts from the superficial vessel [19]. While FAZ area and perimeter are the most stable measures, SFD and SVD measures may be more sensitive to small changes, suggested by the more consistent differences between right and left eyes using the SFD and SVD analyses compared to FAZ analyses.

Our ICC results for SVD and SFD in the SVP are lower than in previous studies: 0.73 in the superficial capillary plexus (Optovue RTVue XR 100 AVANTI—sample size of 70 subjects) [26]; 0.87 in the SVP (Angiovue RTVue-XR—sample size of 27 healthy subjects) [27]; and 0.73 for fractal dimension in the superficial vessels (DRI OCT Triton—sample size of 33 subjects) [28]. Vessel size is disregarded by skeletonisation in SVD, which tends to lower ICC [29]. Differences compared to the reported literature could also be due to segmentation of scans between the different devices used in the studies and a possible reduction in scan quality in the ITU compared to the clinic environment. Our findings of greater ICC for SVD measurements in the superficial vasculature than the deeper vasculature are consistent with previous studies [30]. 

Our data showed better reproducibility of repeated FAZ measurements than blood flow metrics, in agreement with a previous study that showed the FAZ area had a higher repeatability in both the superficial and deep capillary plexus than vessel density [26]. The arrangement of vessels varies between the retinal layers, with vessels in the SVP appearing as radially organised large vessels with interconnected capillaries centred on the FAZ, whereas the ICP and deep capillary plexus (DCP) have finer capillaries arranged in a more dense and complex structure surrounding the FAZ [31], which makes the boundary easier to visualise and therefore measure. 

Previous studies have measured the FAZ area using the SVP and DCP because of projection artefact limiting visualisation of the ICP, with results showing more reliable quantification in the SVP [32]. The recent developments in technology act to reduce image artefact in OCTA, thus allowing more reliable identification of the ICP [31]. Our data show the highest FAZ ICC in the ICP, as FAZ demarcation is most repeatable in this vascular layer, and it is possible that projection artefact from the SVP may make the FAZ in the ICP more prominent. 

Consistent with our results of left–right eye differences, Liu et al. [33] found higher vascular density in right eyes in both superficial and deep retinal capillary networks in a group of 87 healthy individuals using OCTA, but found no difference between the superficial retinal vascular FAZ area of left and right eyes. They suggested that this finding of left–right eye differences related to ocular dominance, with most of their recruited patients being right-eye dominant [33]. It may therefore be beneficial to assess ocular dominance in future work. 

Differences in blood flow between eyes could also relate to left–right differences in vascular anatomy, although previous studies in healthy participants have given varied results, with no difference in perfusion between eyes detected using scanning laser Doppler flowmetry [34] or laser speckle flowgraphy in healthy individuals [35]. However, a study using the Retinal Functional Imager to measure retinal blood flow velocity in 27 normal participants found weak evidence that the average arterial and venous blood flow velocity was faster in the right eye than the left [36]. 

In the posterior cerebral circulation, flow velocity and volume measured by Doppler sonography were greater in the left vertebral artery than the right (*p* < 0.05 for both) in a study size of 180 healthy volunteers [37]. However, in the anterior circulation, cross-sectional blood flow and artery size, assessed by doppler ultrasound, computed tomography and magnetic resonance angiography in a sample size of 152 live adult patient brains, and by digital Vernier calliper measurements in 51 adult donated brains dissected out from human bodies, did not differ between right and left common carotid, anterior, middle, and posterior cerebral arteries [38]. 

Consistent with the difference in blood flow between eyes, we found thinner GCL in left eyes than in right eyes. This is consistent with previous studies, such as Mwanza et al. [39], who used spectral domain OCT (Cirrus HD-OCT) with 284 healthy subjects to show the right eyes had a significantly thicker RNFL than the left eyes and concluded that interocular difference in average RNFL thickness of healthy individuals should not exceed 9 µm when using this particular OCT device. An earlier study using Stratus OCT3 on 103 healthy volunteers found the RNFL of the right eye was 1.2–1.3 µm thicker than the left eye and concluded that the mean RNFL thickness in healthy individuals should not exceed a difference of 9–12 µm between eyes [40]. Because differences in retinal blood flow reflect differences in retinal structure across a variety of pathologies, including optic neuropathies, it is possible that this left–right difference in blood flow reflects the structural asymmetry [3].

We found strong evidence that the global RNFL thickness measure increased in the post-op scans compared to the pre-op scan, while there was no change between these timepoints in the macular structural or blood flow measures. The optic nerve head may be affected by local forces, such as intraocular pressure (IOP) and cerebrospinal fluid pressure [41]. A recent study used OCT to show that the neuroretinal rim thins during the day in healthy individuals, consistent with our finding of increased global RNFL thickness in supine subjects in the ITU. However, in a different study, when subjects shifted from a seated to a head down tilt position, IOP increased but neuroretinal rim thinning did not occur, although head down tilt was only maintained for 3 h [42], while our cohort were supine for a prolonged period, nearing 48 h by the time of the first post-op assessment at scan 2. We suggest that the postural change to the supine position may have changed the effect of gravity on the retrobulbar cerebrospinal fluid dynamics, which may be comparable to the changes seen in space-associated neuro-ocular syndrome [43]. Unfortunately, as a result of time constraints with the patients in the ITU, it was not possible to assess optic nerve head blood flow, and we also did not assess optic nerve head volume on structural scans. It would be beneficial to explore optic nerve head changes associated with prolonged supine posture in future work.

There are other limitations to our study, with the main being the small sample size of 18 participants. As there were no a priori data in our cohort, we did not perform a power calculation; however, the fact that we had the sensitivity to detect left–right eye differences and increased RNFL thickness in the supine imaging position suggests that we were sufficiently powered to detect small effects. The limited available data at the 7 day time point may mean that we would be unable to detect a difference between the 48 h and 7 day timepoints. We did not design the study to compare the tabletop and flex devices, although other work suggests that measures do not differ between the two (unpublished data). There were no patients with known neurodegenerative or neurological disorders included in the study cohort, so the results cannot be applied to patients with these disorders. Future work could include these patients. 

Our study was designed to investigate the stability of retinal structure and blood flow measures between portable OCT and OCTA scans taken pre-operatively in a standard clinical setting and post-operatively in the ITU. Our detection of a small variance in retinal blood flow measures in the ICP between the left and right eyes is consistent with previous reports and, coupled with the fact that the measure has some inter-subject variability, suggests that OCTA performed in the ITU may be sensitive to small changes and therefore able to detect physiological changes associated with microvascular compromise in sepsis.

## 5. Conclusions

OCT and OCTA scans taken in the ITU have the potential to both sensitively and reproducibly study retinal and OCT manifestations of systemic disease and for these measurements to be assessed as biomarkers of disease progression. Furthermore, the physiological stability of these retinal measures suggests their potential to be a clinically useful biomarker in this cohort. 

## Figures and Tables

**Table 1:** Mean patient difference values between timepoints in each blood flow measure of the superficial vascular plexus and intermediate capillary plexus. Timepoints: 1, pre-op; 2 and 3, 24–48 h and 7 days post-op; FAZ: foveal avascular zone.

Retinal Vessel Layer	Measure	Timepoint	Mean	95% Confidence Interval	Standard Error	*p* Value
Superficial vascular plexus	Skeletal fractal dimension	1	1.602	1.584 to 1.621	0.009	0.08
		2 and 3	1.625	1.608 to 1.642	0.008	
	Skeletal vessel density	1	0.068	0.063 to 0.074	0.003	0.29
		2 and 3	0.072	0.067 to 0.077	0.002	
	FAZ area (mm^2^)	1	0.433	0.405 to 0.461	0.014	0.20
		2 and 3	0.408	0.382 to 0.434	0.013	
	FAZ perimeter (mm)	1	2.678	2.565 to 2.791	0.056	0.53
		2 and 3	2.628	2.525 to 2.732	0.052	
Intermediate capillary plexus	Skeletal fractal dimension	1	1.603	1.582 to 1.624	0.010	0.211
		2 and 3	1.621	1.603 to 1.640	0.009	
	Skeletal vessel density	1	0.067	0.062 to 0.072	0.003	0.98
		2 and 3	0.067	0.063 to 0.072	0.002	
	FAZ area (mm^2^)	1	0.271	0.256 to 0.285	0.007	0.06
		2 and 3	0.252	0.239 to 0.265	0.006	
	FAZ perimeter (mm)	1	2.294	2.201 to 2.387	0.046	0.41
		2 and 3	2.242	2.158 to 2.325	0.042	

**Table 2:** Intraclass correlation coefficient of the superficial vascular plexus and intermediate capillary plexus blood flow measures, with the corresponding 95% confidence interval. OS: left eye; OD: right eye; FAZ: foveal avascular zone.

Retinal Vessel Layer	Measure	Eye	Intraclass Correlation Coefficient	95% Confidence Interval
Superficial vascular plexus	Skeletal fractal dimension	OS	0.30	0.06 to 0.56
		OD	0.22	−0.01 to 0.49
	Skeletal vessel density	OS	0.47	0.23 to 0.69
		OD	0.33	0.08 to 0.59
	FAZ area	OS	0.86	0.75 to 0.93
		OD	0.81	0.67 to 0.91
	FAZ perimeter	OS	0.85	0.73 to 0.92
		OD	0.62	0.39 to 0.80
Intermediate capillary plexus	Skeletal fractal dimension	OS	0.08	−0.13 to 0.35
		OD	0.06	−0.14 to 0.35
	Skeletal vessel density	OS	0.33	0.09 to 0.59
		OD	0.12	−0.10 to 0.41
	FAZ area	OS	0.88	0.78 to 0.94
		OD	0.92	0.85 to 0.96
	FAZ perimeter	OS	0.87	0.77 to 0.94
		OD	0.90	0.82 to 0.95

**Table 3:** The intraclass correlation coefficient of the ganglion cell layer and retinal nerve fibre layer thicknesses, with the corresponding 95% confidence interval. OS: left eye; OD: right eye.

Retinal Layer	Measure	Eye	Intraclass Correlation Coefficient	95% Confidence Interval
Retinal nerve fibre layer	Nasal superior	OS	0.97	0.94 to 0.98
		OD	0.97	0.94 to 0.99
	Nasal	OS	0.87	0.75 to 0.94
		OD	0.88	0.78 to 0.94
	Nasal inferior	OS	0.76	0.60 to 0.88
		OD	0.96	0.92 to 0.98
	Temporal inferior	OS	0.96	0.92 to 0.98
		OD	0.98	0.95 to 0.99
	Temporal	OS	0.95	0.92 to 0.98
		OD	0.98	0.95 to 0.99
	Temporal superior	OS	0.99	0.98 to 0.99
		OD	0.81	0.68 to 0.91
	Global	OS	0.96	0.91 to 0.98
		OD	0.98	0.95 to 0.99
Ganglion cell layer	Outer superior	OS	0.90	0.68 to 0.96
		OD	0.93	0.86 to 0.96
	Inner superior	OS	0.61	0.39 to 0.78
		OD	0.81	0.67 to 0.91
	Outer nasal	OS	0.82	0.69 to 0.91
		OD	0.94	0.88 to 0.97
	Inner nasal	OS	0.60	0.38 to 0.78
		OD	0.87	0.76 to 0.93
	Outer inferior	OS	0.78	0.63 to 0.89
		OD	0.91	0.84 to 0.96
	Inner inferior	OS	0.72	0.55 to 0.86
		OD	0.76	0.59 to 0.88
	Outer temporal	OS	0.83	0.70 to 0.92
		OD	0.83	0.70 to 0.92
	Inner temporal	OS	0.55	0.33 to 0.75
		OD	0.67	0.47 to 0.83
	Central	OS	0.93	0.88 to 0.97
		OD	0.82	0.69 to 0.91

**Table 4:** Blood flow measures and inter-eye differences. FAZ: foveal avascular zone; OS: left eye; OD: right eye.

Vascular Plexus	Blood Flow Measure	Eye	Mean Value ± Standard Error	OS/OD Difference	*p* Value
Superficial vascular plexus	Skeletal fractal dimension	OS	1.542 ± 0.024	0.022	0.08
		OD	1.564 ± 0.012		
	Skeletal vessel density	OS	0.049 ± 0.007	0.005	0.14
		OD	0.054 ± 0.004		
	FAZ area	OS	0.344 ± 0.040	0.016	0.40
		OD	0.328 ± 0.019		
	FAZ perimeter	OS	2.425 ± 0.162	0.038	0.62
		OD	2.387 ± 0.076		
Intermediate capillary plexus	Skeletal fractal dimension	OS	1.545 ± 0.027	0.028	0.05
		OD	1.573 ± 0.014		
	Skeletal vessel density	OS	0.051 ± 0.007	0.006	0.07
		OD	0.057 ± 0.003		
	FAZ area	OS	0.195 ± 0.020	0.011	0.06
		OD	0.184 ± 0.010		
	FAZ perimeter	OS	2.044 ± 0.129	0.167	0.01
		OD	1.877 ± 0.062		

**Table 5:** Retinal layer thicknesses and inter-eye differences. OS: left eye; OD: right eye.

Retinal Layer	Location	Eye	Mean Thickness (μm) ± Standard Error	OS/OD Difference (μm)	*p* Value
Retinal nerve fibre layer	Nasal superior	OS	139.759 ± 5.469	10.474	0.0004
		OD	129.285 ± 2.771		
	Nasal	OS	79.998 ± 2.832	2.816	0.054
		OD	82.814 ± 1.435		
	Nasal inferior	OS	138.714 ± 6.188	1.711	0.587
		OD	137.003 ± 3.136		
	Temporal superior	OS	138.219 ± 5.808	8.790	0.004
		OD	147.009 ± 2.943		
	Temporal	OS	68.864 ± 1.839	2.737	0.005
		OD	71.601 ± 0.932		
	Temporal inferior	OS	148.000 ± 4.418	3.290	0.147
		OD	144.710 ± 2.239		
	Global	OS	107.942 ± 1.270	0.368	0.569
		OD	108.310 ± 0.643		
Macular ganglion cell layer	Outer superior	OS	27.442 ± 0.957	1.032	0.033
		OD	28.474 ± 0.474		
	Inner superior	OS	44.600 ± 1.516	0.710	0.348
		OD	45.310 ± 0.751		
	Outer nasal	OS	31.320 ± 1.087	1.738	0.002
		OD	29.582 ± 0.538		
	Inner nasal	OS	42.193 ± 1.800	1.499	0.097
		OD	43.692 ± 0.891		
	Outer inferior	OS	28.054 ± 1.088	0.089	0.869
		OD	27.965 ± 0.539		
	Inner inferior	OS	45.432 ± 1.635	0.844	0.301
		OD	44.588 ± 0.810		
	Outer temporal	OS	31.834 ± 1.291	0.771	0.233
		OD	32.605 ± 0.640		
	Inner temporal	OS	43.139 ± 1.600	1.222	0.128
		OD	41.917 ± 0.793		
	Central	OS	20.244 ± 1.047	0.690	0.188
		OD	19.554 ± 0.518		

## Data Availability

Data are available upon reasonable request.

## Supplementary Materials

The following are available online at https://www.mdpi.com/article/10.3390/diagnostics11081516/s1; Figure S1: Superficial vascular plexus and intermediate capillary plexus patient timepoint effect mean graphs, Figure S2: Retinal nerve fibre layer thickness—timepoint effect mean graphs, Figure S3: Ganglion cell layer thickness—timepoint effect mean graphs, Table S1a: Extent of scan completion in participants, Table S1b: Haemodynamic parameters of participants, Table S2: Mean difference between timepoints (1, pre-op; 2 and 3, 24 h and 7 days post-op) of the retinal nerve fibre layer and ganglion cell layer thicknesses (measured in μm), Table S3: Summary of vessel layer densities, including standard deviation (SD), Table S4: Summary of retinal structural layer thicknesses.

## Abbreviations

OCT: optical coherence tomography; RNFL: retinal nerve fibre layer; ONH: optic nerve head; OCTA; optical coherence tomography angiography; ITU: intensive therapy unit; pre-op: pre-operative; post-op: post-operative; ART: automatic real time; SVP: superficial vascular plexus; ICP: intermediate capillary plexus; SFD: skeletal fractal dimension; SVD: skeletonised vessel density; FAZ: foveal avascular zone; GCL: ganglion cell layer; OS: left eye; OD: right eye; ICC: intraclass correlation coefficient; ETDRS: Early Treatment Diabetic Retinopathy Study; DCP: deep capillary plexus; IOP: intraocular pressure.

## References

[1] Adhi M., Duker J.S. (2013). Optical coherence tomography--current and future applications. Curr. Opin. Ophthalmol..

[2] Kashani A.H., Chen C.-L., Gahm J.K., Zheng F., Richter G.M., Rosenfeld P.J., Shi Y., Wang R.K. (2017). Optical coherence tomography angiography: A comprehensive review of current methods and clinical applications. Prog. Retin. Eye Res..

[3] Courtie E., Veenith T., Logan A., Denniston A.K., Blanch R.J. (2020). Retinal blood flow in critical illness and systemic disease: A review. Ann. Intensive Care.

[4] Kwapong W.R., Ye H., Peng C., Zhuang X., Wang J., Shen M., Lu F. (2018). Retinal Microvascular Impairment in the Early Stages of Parkinson’s Disease. Investig. Opthalmol. Vis. Sci..

[5] Lad E.M., Mukherjee D., Stinnett S.S., Cousins S.W., Potter G.G., Burke J.R., Farsiu S., Whitson H.E. (2018). Evaluation of inner retinal layers as biomarkers in mild cognitive impairment to moderate Alzheimer’s disease. PLoS ONE.

[6] Querques G., Borrelli E., Sacconi R., De Vitis L., Leocani L., Santangelo R., Magnani G., Comi G., Bandello F. (2019). Functional and morphological changes of the retinal vessels in Alzheimer’s disease and mild cognitive impairment. Sci. Rep..

[7] Patton N., Aslam T., Macgillivray T., Pattie A., Deary I.J., Dhillon B. (2005). Retinal vascular image analysis as a potential screening tool for cerebrovascular disease: A rationale based on homology between cerebral and retinal microvasculatures. J. Anat..

[8] Alnawaiseh M. (2019). Evaluation of ocular perfusion in patients with atrial fibrillation using optical coherence tomography angiography. Invest. Ophthalmol. Vis. Sci..

[9] Arnould L., Guenancia C. (2018). The EYE-MI Pilot Study: A Prospective Acute Coronary Syndrome Cohort Evaluated With Retinal Optical Coherence Tomography Angiography. Investig. Opthalmol. Vis. Sci..

[10] Terheyden J.H. (2019). Impaired retinal capillary perfusion assessed by optical coherence tomography angiography in patients with recent systemic hypertensive crisis. Investig. Ophthalmol. Vis. Sci..

[11] Nakayama L. (2019). Retinal Avascular Foveal Zone as an systemic biomarker to evaluate inflammatory bowel disease control. Invest. Ophthalmol. Vis. Sci..

[12] Park J. (2016). Microcirculatory alterations in hemorrhagic shock and sepsis with optical coherence tomography. Crit. Care Med..

[13] Alnawaiseh M., Ertmer C., Seidel L., Arnemann P.H., Lahme L., Kampmeier T.-G., Rehberg S.W., Heiduschka P., Eter N., Hessler M. (2018). Feasibility of optical coherence tomography angiography to assess changes in retinal microcirculation in ovine haemorrhagic shock. Crit. Care.

[14] Lin B.R. (2019). Characterizing changes in retinal perfusion in high risk pregnancies with optical coherence tomography angiography. Invest. Ophthalmol. Vis. Sci..

[15] Helmy O. (2019). Detection of early retinal vascular structural changes of pre-eclamptic patients using optical coherence tomography angiography. Invest. Ophthalmol. Vis. Sci..

[16] Bäckman C.G., Ahlberg M. (2018). Group meetings after critical illness—Giving and receiving strength. Intensive Crit. Care Nurs..

[17] Aliaga M., Forel J.-M., De Bourmont S., Jung B., Thomas G., Mahul M., Bisbal M., Nougaret S., Hraiech S., Roch A. (2015). Diagnostic yield and safety of CT scans in ICU. Intensive Care Med..

[18] Liu X., Kale A.U., Capewell N., Talbot N., Ahmed S., Keane P.A., Mollan S., Belli A., Blanch R.J., Veenith T. (2019). Optical coherence tomography (OCT) in unconscious and systemically unwell patients using a mobile OCT device: A pilot study. BMJ Open.

[19] Tan A.C.S. (2018). An overview of the clinical applications of optical coherence tomography angiography. Eye.

[20] Zahid S., Dolz-Marco R., Freund K.B., Balaratnasingam C., Dansingani K., Gilani F., Mehta N., Young E., Klifto M.R., Chae B. (2016). Fractal dimensional analysis of optical coherence tomography angiography in eyes with diabetic retinopathy. Investig. Ophthalmol. Vis. Sci..

[21] Schindelin J., Arganda-Carreras I., Frise E., Kaynig V., Longair M., Pietzsch T., Preibisch S., Rueden C., Saalfeld S., Schmid B. (2012). Fiji: An open-source platform for biological-image analysis. Nat. Methods.

[22] Shrout P.E., Fleiss J.L. (1979). Intraclass correlations: Uses in assessing rater reliability. Psychol. Bull..

[23] R Core Team R: A Language and Environment for Statistical Computing.

[24] Wickham H. (2016). ggplot2: Elegant Graphics for Data Analysis.

[25] Revelle W. (2020). psych: Procedures for Psychological, Psychometric, and Personality Research. https://cran.r-project.org/web/packages/psych/psych.pdf.

[26] Coscas F., Sellam A., Glacet-Bernard A., Jung C., Goudot M., Miere A., Souied E.H. (2016). Normative data for vascular density in superficial and deep capillary plexuses of healthy adults assessed by optical coherence tomography angiography. Investig. Ophthalmol. Vis. Sci..

[27] Venugopal J.P., Rao H.L., Weinreb R.N., Pradhan Z.S., Dasari S., Riyazuddin M., Puttiah N.K., Rao D.A.S., Devi S., Mansouri K. (2018). Repeatability of vessel density measurements of optical coherence tomography angiography in normal and glaucoma eyes. Br. J. Ophthalmol..

[28] Fang D., Tang F.Y., Huang H., Cheung C.Y., Chen H. (2019). Repeatability, interocular correlation and agreement of quantitative swept-source optical coherence tomography angiography macular metrics in healthy subjects. Br. J. Ophthalmol..

[29] Eastline M., Munk M.R., Wolf S., Schaal K.B., Ebneter A., Tian M., Giannakaki-Zimmermann H., Zinkernagel M.S. (2019). Repeatability of wide-field optical coherence tomography angiography in normal retina. Transl. Vis. Sci. Technol..

[30] Al-Sheikh M., Tepelus T.C., Nazikyan T., Sadda S.R. (2017). Repeatability of automated vessel density measurements using optical coherence tomography angiography. Br. J. Ophthalmol..

[31] Garrity S.T., Iafe N.A., Phasukkijwatana N., Chen X., Sarraf D. (2017). Quantitative analysis of three distinct retinal capillary plexuses in healthy eyes using optical coherence tomography angiography. Investig. Ophthalmol. Vis. Sci..

[32] Shahlaee A., Pefkianaki M., Hsu J., Ho A.C. (2016). Measurement of Foveal Avascular Zone Dimensions and its Reliability in Healthy Eyes Using Optical Coherence Tomography Angiography. Am. J. Ophthalmol..

[33] Liu G., Keyal K., Wang F. (2017). Interocular Symmetry of Vascular Density and Association with Central Macular Thickness of Healthy Adults by Optical Coherence Tomography Angiography. Sci. Rep..

[34] Rawji M., Flanagan J. (2001). Intraocular and interocular symmetry in normal retinal capillary perfusion. J. Glaucoma.

[35] Shinohara Y., Kashima T., Akiyama H., Shimoda Y., Li D., Kishi S. (2017). Evaluation of Fundus Blood Flow in Normal Individuals and Patients with Internal Carotid Artery Obstruction Using Laser Speckle Flowgraphy. PLoS ONE.

[36] Landa G., Jangi A.A., Garcia P.M.T., Rosen R.B. (2012). Initial report of quantification of retinal blood flow velocity in normal human subjects using the Retinal Functional Imager (RFI). Int. Ophthalmol..

[37] Albayrak R., Degirmenci B., Acar M., Haktanir A., Colbay M., Yaman M. (2007). Doppler sonography evaluation of flow velocity and volume of the extracranial internal carotid and vertebral arteries in healthy adults. J. Clin. Ultrasound.

[38] Burlakoti A., Kumaratilake J., Taylor J., Henneberg M. (2019). Asymmetries of total arterial supply of cerebral hemispheres do not exist. Heliyon.

[39] Mwanza J.C., Durbin M.K., Budenz D.L. (2011). Interocular symmetry in peripapillary retinal nerve fiber layer thickness measured with the cirrus HD-OCT in healthy eyes. Am. J. Ophthalmol..

[40] Budenz D.L. (2008). Symmetry between the right and left eyes of the normal retinal nerve fiber layer measured with optical coherence tomography (an AOS thesis). Trans. Am. Ophthalmol. Soc..

[41] Hua Y., Voorhees A.P., Sigal I.A. (2018). Cerebrospinal fluid pressure: Revisiting factors influencing optic nerve head biomechanics. Investig. Ophthalmol. Vis. Sci..

[42] Pardon L.P., Cheng H., Chettry P., Patel N.B. (2020). Optic nerve head morphological changes over 12 hours in seated and head-down tilt postures. Investig. Ophthalmol. Vis. Sci..

[43] Lee A.G., Mader T.H., Gibson C.R., Tarver W., Rabiei P., Riascos R.F., Galdamez L.A., Brunstetter T. (2020). Spaceflight associated neuro-ocular syndrome (SANS) and the neuro-ophthalmologic effects of microgravity: A review and an update. NPJ Microgravity.

