# Supplementary material for: Stability of OCT and OCTA in the Intensive Therapy Unit Setting
Source: Diagnostics (Basel). 2021 Aug 23;11(8):1516. doi: 10.3390/diagnostics11081516 (PMC8394026; doi:10.3390/diagnostics11081516)
Supplement: Supplementary file 1 [file diagnostics-11-01516-s001.zip › diagnostics-1296937-supplementary.pdf]

**Stability of OCT and OCTA in the intensive therapy unit setting.**

Ella F. Courtie[1, 2, 3], Aditya U. Kale[2], Benjamin TK. Hui[2], Xiaoxuan Liu[2, 4, 5, 6], Nicholas I. Capewell[2], Jonathan RB. Bishop[3], Tony Whitehouse[7, 8], Tonny Veenith[7, 8], Ann Logan[9, 10], Alastair K. Denniston[2, 4, 11, 12], Richard J. Blanch[1, 2, 3, 13].

[1]Neuroscience and Ophthalmology, Institute of Inflammation and Ageing, College of Medical and Dental Sciences, University of Birmingham, UK

[2]Ophthalmology Department, University Hospitals Birmingham NHS Foundation Trust, Birmingham, UK

[3]NIHR Surgical Reconstruction and Microbiology Research Centre, University Hospitals Birmingham NHS Foundation Trust, Birmingham, UK

[4]Academic Unit of Ophthalmology, Institute of Inflammation and Ageing, College of Medical and Dental Sciences, University of Birmingham, UK

[5]Health Data Research UK, London, UK

[6]Moorfields Eye Hospital NHS Foundation Trust, UK

[7]Critical Care Unit, University Hospitals Birmingham NHS Foundation Trust, Birmingham, UK

[8]Birmingham Acute Care Research Group, Institute of Inflammation and Ageing, College of Medical and Dental Sciences, University of Birmingham, UK

[9]Axolotl Consulting Ltd, Droitwich, Worcestershire, WR9 0JS, UK

[10]Division of Biomedical Sciences, Warwick Medical School, University of Warwick, Coventry, CV4 7HL, UK

[11]NIHR Biomedical Research Centre for Ophthalmology, Moorfields Eye Hospital NHS Foundation Trust and UCL Institute of Ophthalmology, London, UK

[12]Centre for Regulatory Science and Innovation, Birmingham Health Partners, UK

[13]Academic Department of Military Surgery and Trauma, Royal Centre for Defence Medicine, Birmingham, UK

Corresponding author: Lt Col Richard Blanch, Institute of Inflammation and Ageing, College of Medical and Dental Sciences, University of Birmingham, United Kingdom.

Email: r.j.blanch@bham.ac.uk

# Superficial vascular plexus

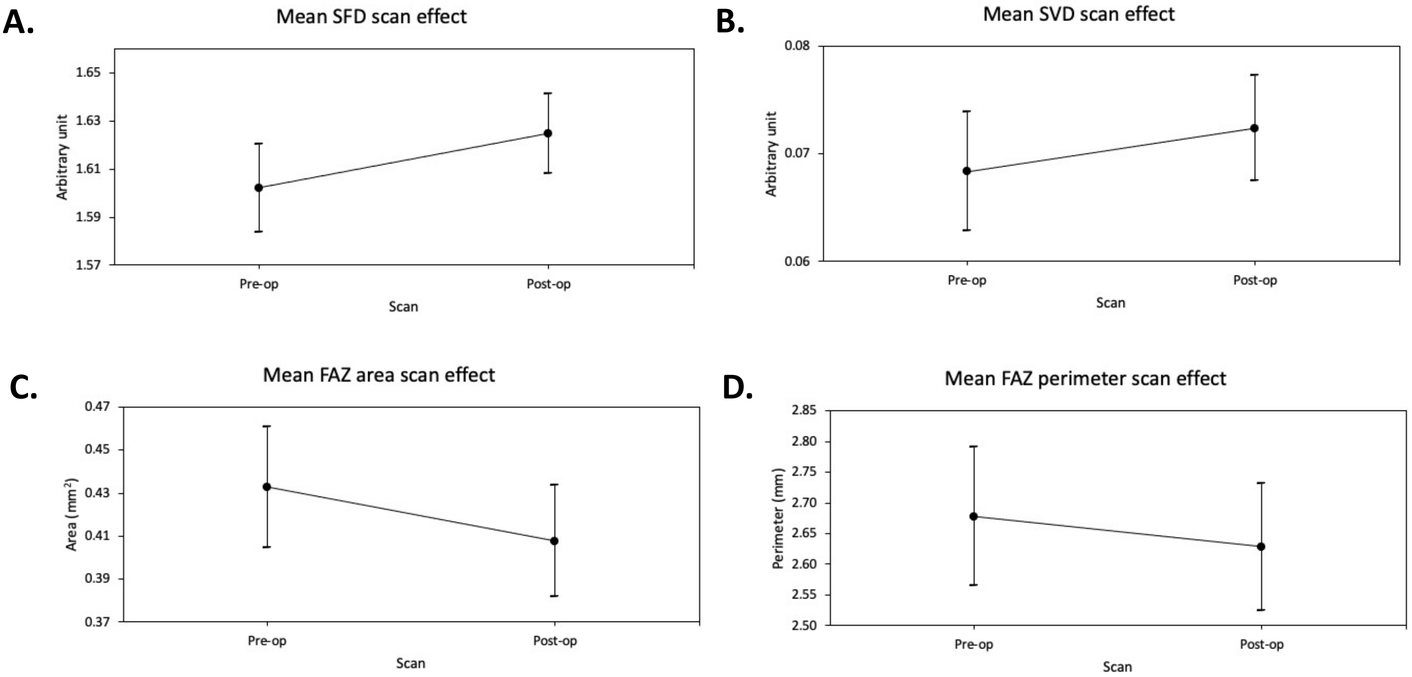


# Intermediate capillary plexus

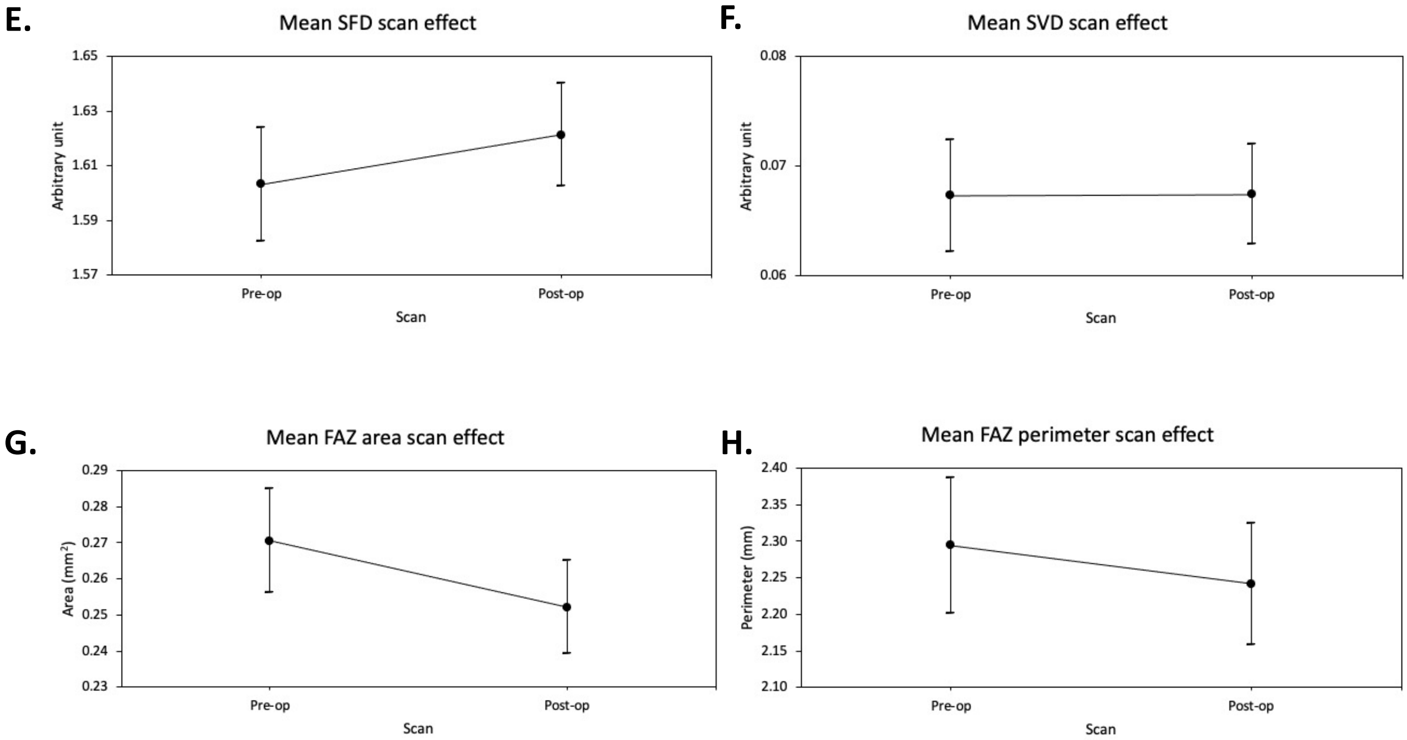


**Figure S1.** Superficial vascular plexus and intermediate capillary plexus patient timepoint effect mean graphs.
Mean difference between scan time points for each measure, with no evidence of a systematic difference between timepoints. Error bars show the 95% confidence interval upper and lower limits.
**A.** Mean difference in skeletal fractal dimension (SFD) of the superficial vascular plexus (SVP) between scans. **B.** Mean difference in skeletal vessel density (SVD) of the SVP between scans. **C.** Mean difference in foveal avascular zone (FAZ) area (measured in $mm^2$) of the SVP between scans. **D.** Mean difference in FAZ perimeter (measured in mm) of the SVP between scans. **E.** Mean difference in SFD of the intermediate capillary plexus (ICP) between scans. **F.** Mean difference in SVD of the ICP between scans. **G.** Mean difference in the FAZ area (measured in $mm^2$) of the ICP between scans. **H.** Mean difference in the FAZ perimeter (measured in mm) of the ICP between scans. Abbreviations. SVP: superficial vascular plexus; SFD: skeletal fractal dimensions; SVD: skeletal vessel density; FAZ: foveal avascular zone; ICP: intermediate capillary plexus.

# Retinal nerve fibre layer

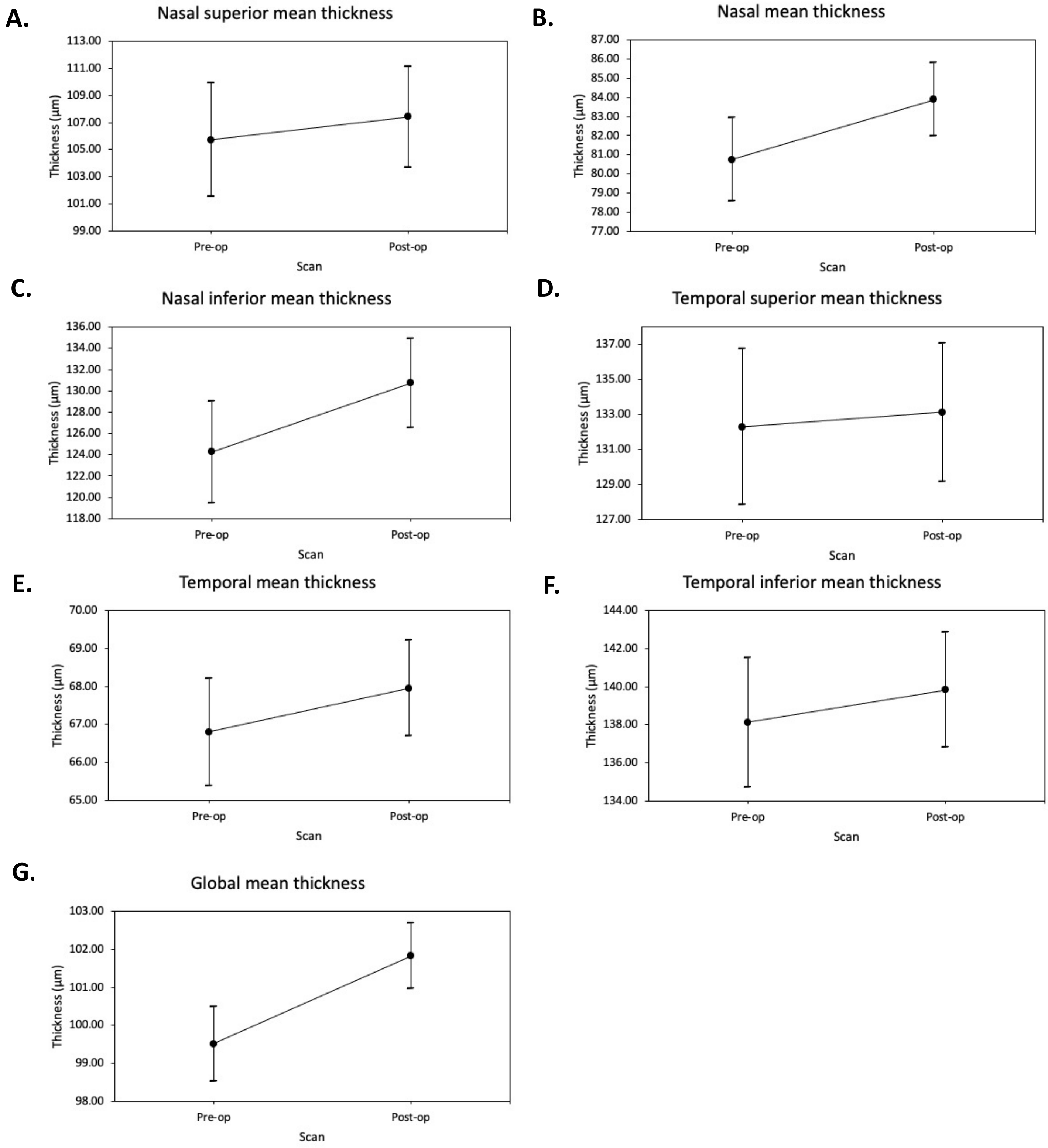


**Figure S2.** Retinal nerve fibre layer thickness - timepoint effect mean graphs.
These graphs show the mean patient difference between each scan timepoint taken measuring the retinal nerve fibre layer (RNFL) thickness, in all 7 segments. All thicknesses are measured in *µm*. Error bars show the 95% confidence interval upper and lower limits.
**A.** Mean difference in RNFL nasal superior thickness between timepoints. **B.** Mean difference in RNFL nasal thickness between scans. **C.** Mean difference in RNFL nasal inferior thickness between timepoints. **D.** Mean difference in RNFL temporal superior thickness between timepoints. **E.** Mean difference in RNFL temporal thickness between timepoints. **F.** Mean difference in RNFL temporal inferior thickness between timepoints. **G.** Mean difference in RNFL global thickness between timepoints.

# Ganglion cell layer

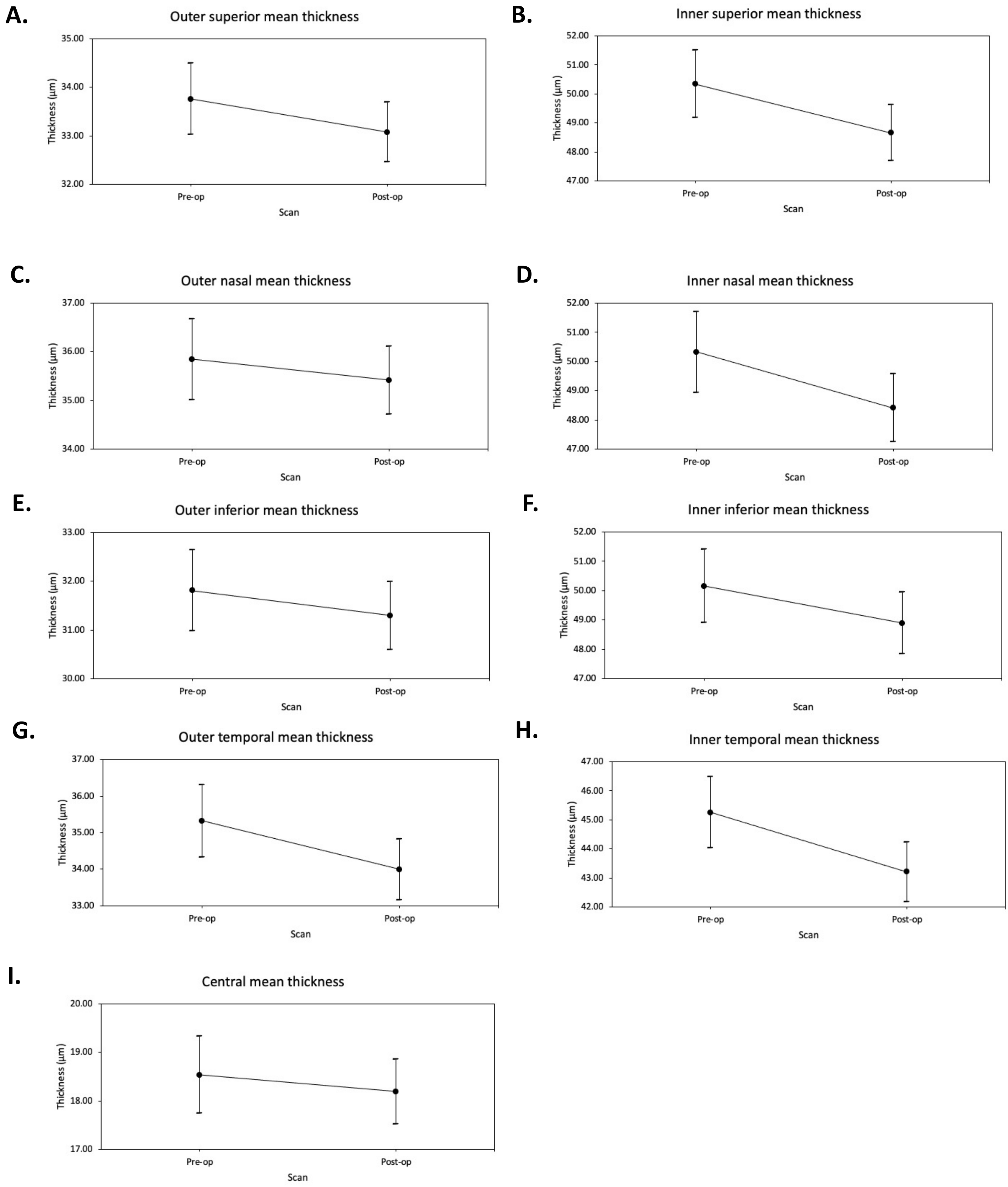


**Figure S3.** Ganglion cell layer thickness - timepoint effect mean graphs.
Mean patient difference between each scan taken measuring the ganglion cell layer (GCL) thickness, in all 9 segments. All thicknesses are measured in *µm*. Error bars show the 95% confidence interval upper and lower limits.
**A.** Mean difference in GCL outer superior thickness between timepoints. **B.** Mean difference in GCL inner superior thickness between timepoints. **C.** Mean difference in GCL outer nasal thickness between timepoints. **D.** Mean difference in GCL inner nasal thickness between timepoints. **E.** Mean difference in GCL outer inferior thickness between timepoints. **F.** Mean difference in GCL inner inferior thickness between timepoints. **G.** Mean difference in GCL outer temporal thickness between timepoints. **H.** Mean difference in GCL inner temporal thickness between timepoints. **I.** Mean difference in GCL central thickness between timepoints.

**Table S1a. Extent of scan completion in participants.** Abbreviations. OCT: optical coherence tomography; OCTA: optical coherence tomography angiography; OD: right eye; RNF: retinal nerve fibre layer.

| Participant | Pre-op scans | 24-48 hours post-op scans | 7 day post-op scans | Comments |
|---|---|---|---|---|
| O1 | Well-tolerated, all scans obtained. Not dilated. | Well-tolerated, all scans obtained. Dilated. | Well-tolerated, all scans obtained. Dilated. | Uncomplicated post-op course. |
| O2 | Well-tolerated, all scans obtained. | Difficulty obtaining scans due to fatigue causing poor fixation. Dilated. | Well-tolerated, all scans obtained with patient supine with pillows supporting their head. Dilated. | Uncomplicated post-op course. |
| O3 | Well-tolerated, all scans obtained. | OCT scans obtained but unable to collect OCTA scans due to fatigue. Dilated. | Well-tolerated, all scans obtained with patient semi-recumbent. | Uncomplicated post-op course. |
| O4 | Well-tolerated, all scans obtained. | Well-tolerated, all scans obtained with patient supine in bed with pillows supporting their head. Not dilated. | Well-tolerated, all scans obtained with patient semi-recumbent. | Uncomplicated post-op course. |
| O5 | Well-tolerated, all scans obtained. | Only OD OCTA obtained due to patient experiencing pain. Dilated. | Well-tolerated, all scans obtained with patient semi-recumbent. Not dilated. | Uncomplicated post-op course. |
| O6 | Well-tolerated, all scans obtained. | Some difficulty obtaining scans. Patient sitting in a chair with head unsupported. | Well-tolerated, all scans obtained with patient seated in a chair. | Uncomplicated post-op course. |
| O7 | Well-tolerated, all scans obtained. | Well-tolerated, all scans obtained. Not dilated. | Unable to image as patient was discharged. | Uncomplicated post-op course. |
| O8 | Well-tolerated, all scans obtained with patient semi-recumbent. | Well-tolerated, all scans obtained with patient semi-recumbent. Dilated. | Unable to image as patient was discharged. | Uncomplicated post-op course. |
| O9 | Well-tolerated, all scans obtained but poor fixation. | Only OCT macula and RNFL obtained, due to patient experiencing pain and fatigue. Patient was seated. Dilated. | Only macula scan obtained. Dilated. | Uncomplicated post-op course. |
| O10 | Well-tolerated, all scans obtained but poor fixation. | No scans obtained due to patient fatigue. | No scans obtained. | Uncomplicated post-op course. |
| O11 | Well-tolerated, all scans obtained. | All scans obtained with patient supine in bed with the head supported by pillows. Dilated. | Well-tolerated, all scans obtained with patient seated in a chair. Not dilated. | Uncomplicated post-op course. |
| O12 | Well-tolerated, all scans obtained. | Well-tolerated, all scans obtained. Not dilated. | Well-tolerated, all scans obtained. Not dilated. | Uncomplicated post-op course. |
| O13 | Well-tolerated, all scans obtained. | Well-tolerated, all scans obtained. Dilated. | NA | Uncomplicated post-op course. |
| O14 | Well-tolerated, all scans obtained. | Well-tolerated, all scans obtained. Not dilated. | NA | Uncomplicated post-op course. |
| O15 | Well-tolerated, all scans obtained. | Well-tolerated, all scans obtained. Not dilated. | NA | Uncomplicated post-op course. |
| O16 | Well-tolerated, all scans obtained. | Well-tolerated, all scans obtained. Not dilated. | NA | Uncomplicated post-op course. |
| O17 | Well-tolerated, all scans obtained. | Well-tolerated, all scans obtained. Not dilated. | NA | Uncomplicated post-op course. |
| O18 | Well-tolerated, all scans obtained. | Well-tolerated, all scans obtained. Not dilated. | NA | Uncomplicated post-op course. |

**Table S1b. Haemodynamic parameters of participants.** Abbreviations. BP: blood pressure; HR: heart rate; $pO_2$: partial pressure of oxygen; $FiO_2$: fraction of inspired oxygen

| Participant | 24-48 hour post-op scans | | | | 7 day post-op scans | | |
|---|---|---|---|---|---|---|---|
| | BP (mmHg) | HR (bpm) | $pO_2$ (kPa) | $FiO_2$ | BP (mmHg) | HR (bpm) | $SaO_2$ (%) |
| **O1** | 133/56 | 66 | 9.7 | 0.28 | 118/47 | 85 | 96 |
| **O2** | 119/63 | 79 | 11.5 | 0.40 | 138/88 | 97 | 97 |
| **O3** | 101/62 | 88 | 9.6 | 0.28 | 117/85 | 82 | 95 |
| **O4** | 116/61 | 82 | 11.4 | 0.28 | 132/84 | 84 | 95 |
| **O5** | 122/66 | 83 | 9.0 | 0.70 | 124/64 | 63 | 93 |
| **O6** | 133/57 | 70 | 12.2 | 0.24 | 135/79 | 91 | 96 |
| **O7** | 114/53 | 53 | 13.8 | 0.24 | NA | NA | NA |
| **O8** | 126/58 | 72 | 9.9 | 0.36 | NA | NA | NA |
| **O9** | 124/68 | 65 | 11.3 | 0.50 | 144/76 | 101 | 95 |
| **O10** | 115/57 | 73 | 12.3 | 0.24 | NA | NA | NA |
| **O11** | 120/68 | 80 | 11.7 | 0.25 | 127/84 | 70 | 95 |
| **O12** | 126/68 | 69 | 12.1 | 0.28 | 118/64 | 70 | 95 |
| **O13** | 108/67 | 83 | 10.6 | 0.50 | NA | NA | NA |
| **O14** | 102/59 | 75 | 5.3 | 0.26 | NA | NA | NA |
| **O15** | 130/75 | 78 | 10.7 | 0.36 | NA | NA | NA |
| **O16** | 118/53 | 80 | 9.9 | 0.28 | NA | NA | NA |
| **O17** | 147/93 | 108 | 10.3 | 0.28 | NA | NA | NA |
| **O18** | 124/85 | 73 | 4.8 | 0.21 | NA | NA | NA |

**Table S2.** Mean difference between timepoints (1, pre-op; 2 and 3, 24 h and 7 days post-op) of the retinal nerve fibre layer and ganglion cell layer thicknesses (measured in µm).

| Retinal layer | Segment | Timepoint | Mean thickness (µm) | 95% confidence interval (µm) |
|---|---|---|---|---|
| Retinal nerve fibre layer | Nasal superior | 1<br>2 and 3 | 105.715<br>107.432 | 101.513 to 109.917<br>103.705 to 111.158 |
| | Nasal | 1<br>2 and 3 | 80.744<br>83.885 | 78.568 to 82.920<br>81.956 to 85.815 |
| | Nasal inferior | 1<br>2 and 3 | 124.249<br>130.711 | 119.495 to 129.003<br>126.495 to 134.927 |
| | Temporal superior | 1<br>2 and 3 | 132.284<br>133.115 | 127.823 to 136.746<br>129.158 to 137.072 |
| | Temporal | 1<br>2 and 3 | 66.802<br>67.953 | 65.389 to 68.215<br>66.700 to 69.206 |
| | Temporal inferior | 1<br>2 and 3 | 138.120<br>139.837 | 134.725 to 141.514<br>136.826 to 142.847 |
| | Global | 1<br>2 and 3 | 99.510<br>101.821 | 98.535 to 100.486<br>100.956 to 102.687 |
| Ganglion cell layer | Outer superior | 1<br>2 and 3 | 33.757<br>33.071 | 33.025 to 34.490<br>32.455 to 33.687 |
| | Inner superior | 1<br>2 and 3 | 50.339<br>48.656 | 49.179 to 51.499<br>47.681 to 49.631 |
| | Outer nasal | 1<br>2 and 3 | 35.844<br>35.418 | 35.012 to 36.676<br>34.718 to 36.117 |
| | Inner nasal | 1<br>2 and 3 | 50.318<br>48.405 | 48.941 to 51.696<br>47.247 to 49.563 |
| | Outer inferior | 1<br>2 and 3 | 31.812<br>31.297 | 30.979 to 32.645<br>30.597 to 31.998 |
| | Inner inferior | 1<br>2 and 3 | 50.155<br>48.889 | 48.904 to 51.406<br>47.837 to 49.941 |
| | Outer temporal | 1<br>2 and 3 | 35.323<br>33.994 | 34.335 to 36.311<br>33.163 to 34.825 |
| | Inner temporal | 1<br>2 and 3 | 45.250<br>43.209 | 44.025 to 46.475<br>42.179 to 44.239 |
| | Central | 1<br>2 and 3 | 18.537<br>18.188 | 17.736 to 19.338<br>17.515 to 18.861 |

**Table S3.** Summary of vessel layer densities, including standard deviation (SD). N refers to number of scans included for each eye – left (OS) and right (OD). Timepoints: 1, pre-op; 2, 24 h and 7 days post-op. Abbreviations. FAZ: foveal avascular zone.

| Vessel Layer | Measure | Scan | OS | | | OD | | |
|---|---|---|---|---|---|---|---|---|
| | | | N | Mean | SD | N | Mean | SD |
| Superficial vascular plexus | Skeletal fractal dimension | 1 | 18 | 1.588 | 0.084 | 17 | 1.613 | 0.054 |
| | | 2 | 16 | 1.623 | 0.061 | 15 | 1.638 | 0.042 |
| | | 3 | 6 | 1.583 | 0.076 | 6 | 1.647 | 0.031 |
| | Skeletal density | 1 | 18 | 0.065 | 0.025 | 17 | 0.070 | 0.019 |
| | | 2 | 16 | 0.072 | 0.021 | 15 | 0.077 | 0.019 |
| | | 3 | 6 | 0.058 | 0.022 | 6 | 0.080 | 0.015 |
| | FAZ area ($mm^2$) | 1 | 17 | 0.472 | 0.212 | 17 | 0.421 | 0.167 |
| | | 2 | 15 | 0.415 | 0.147 | 15 | 0.375 | 0.122 |
| | | 3 | 4 | 0.385 | 0.028 | 6 | 0.412 | 0.104 |
| | FAZ perimeter (mm) | 1 | 17 | 2.759 | 0.650 | 17 | 2.649 | 0.440 |
| | | 2 | 15 | 2.651 | 0.583 | 15 | 2.475 | 0.440 |
| | | 3 | 4 | 2.593 | 0.169 | 6 | 2.830 | 0.527 |
| Intermediate capillary plexus | Skeletal fractal dimension | 1 | 18 | 1.589 | 0.086 | 17 | 1.619 | 0.046 |
| | | 2 | 16 | 1.612 | 0.083 | 15 | 1.634 | 0.034 |
| | | 3 | 6 | 1.587 | 0.060 | 6 | 1.647 | 0.025 |
| | Skeletal density | 1 | 18 | 0.064 | 0.023 | 17 | 0.070 | 0.012 |
| | | 2 | 16 | 0.066 | 0.021 | 15 | 0.071 | 0.014 |
| | | 3 | 6 | 0.055 | 0.016 | 6 | 0.076 | 0.012 |
| | FAZ area ($mm^2$) | 1 | 15 | 0.285 | 0.121 | 16 | 0.261 | 0.135 |
| | | 2 | 14 | 0.250 | 0.107 | 14 | 0.234 | 0.111 |
| | | 3 | 4 | 0.277 | 0.072 | 6 | 0.271 | 0.092 |
| | FAZ perimeter (mm) | 1 | 15 | 2.368 | 0.486 | 16 | 2.191 | 0.535 |
| | | 2 | 14 | 2.252 | 0.610 | 14 | 2.102 | 0.526 |
| | | 3 | 4 | 2.638 | 0.516 | 6 | 2.367 | 0.410 |

**Table S4.** Summary of retinal structural layer thicknesses. N refers to number of scans included for each eye – left (OS) and right (OD). Timepoints: 1, pre-op; 2, 24 h and 7 days post-op.

| Retinal Layer | Segment | Scan | OS | | | OD | | |
|---|---|---|---|---|---|---|---|---|
| | | | N | Mean | SD | N | Mean | SD |
| Retinal nerve fibre layer | Nasal superior | 1 | 18 | 107 | 30 | 17 | 98 | 21 |
| | | 2 | 16 | 108 | 31 | 15 | 102 | 22 |
| | | 3 | 7 | 128 | 30 | 6 | 113 | 17 |
| | Nasal | 1 | 18 | 78 | 12 | 17 | 83 | 12 |
| | | 2 | 16 | 83 | 16 | 15 | 87 | 16 |
| | | 3 | 7 | 81 | 17 | 6 | 83 | 15 |
| | Nasal inferior | 1 | 18 | 121 | 32 | 17 | 125 | 26 |
| | | 2 | 16 | 134 | 43 | 15 | 128 | 30 |
| | | 3 | 7 | 134 | 46 | 6 | 130 | 35 |
| | Temporal inferior | 1 | 18 | 140 | 22 | 17 | 133 | 24 |
| | | 2 | 16 | 141 | 26 | 15 | 138 | 28 |
| | | 3 | 7 | 152 | 29 | 6 | 138 | 36 |
| | Temporal | 1 | 18 | 66 | 11 | 17 | 67 | 14 |
| | | 2 | 16 | 66 | 11 | 15 | 68 | 15 |
| | | 3 | 7 | 71 | 11 | 6 | 76 | 13 |
| | Temporal superior | 1 | 18 | 128 | 24 | 17 | 133 | 21 |
| | | 2 | 16 | 129 | 24 | 15 | 133 | 29 |
| | | 3 | 7 | 138 | 16 | 6 | 151 | 14 |
| | Global | 1 | 18 | 98 | 13 | 17 | 99 | 13 |
| | | 2 | 16 | 101 | 14 | 15 | 101 | 14 |
| | | 3 | 7 | 107 | 13 | 6 | 106 | 14 |
| Ganglion cell layer | Outer superior | 1 | 18 | 32.8 | 5.8 | 17 | 33.9 | 4.9 |
| | | 2 | 17 | 33.1 | 4.3 | 16 | 34.0 | 4.6 |
| | | 3 | 8 | 31.4 | 5.2 | 8 | 34.6 | 4.0 |
| | Inner superior | 1 | 18 | 49.8 | 5.0 | 17 | 50.2 | 6.7 |
| | | 2 | 17 | 47.8 | 4.5 | 16 | 49.3 | 6.8 |
| | | 3 | 8 | 47.8 | 9.1 | 8 | 51.5 | 4.5 |
| | Outer nasal | 1 | 18 | 36.3 | 5.0 | 17 | 34.4 | 6.0 |
| | | 2 | 17 | 35.8 | 4.6 | 16 | 34.9 | 5.7 |
| | | 3 | 8 | 36.6 | 6.9 | 8 | 36.5 | 5.0 |
| | Inner nasal | 1 | 18 | 49.7 | 5.8 | 17 | 50.5 | 5.6 |
| | | 2 | 17 | 46.7 | 6.2 | 16 | 49.7 | 6.8 |
| | | 3 | 8 | 48.4 | 6.8 | 8 | 50.5 | 6.2 |
| | Outer inferior | 1 | 18 | 31.4 | 4.3 | 17 | 31.1 | 5.4 |
| | | 2 | 17 | 31.2 | 4.5 | 16 | 31.4 | 5.4 |
| | | 3 | 8 | 31.1 | 6.6 | 8 | 33.8 | 4.4 |
| | Inner inferior | 1 | 18 | 50.1 | 5.0 | 17 | 49.9 | 5.5 |
| | | 2 | 17 | 48.8 | 4.6 | 16 | 48.6 | 8.4 |
| | | 3 | 8 | 50.6 | 4.8 | 8 | 48.6 | 5.6 |
| | Outer temporal | 1 | 18 | 34.4 | 5.2 | 17 | 35.2 | 5.8 |
| | | 2 | 17 | 33.3 | 4.6 | 16 | 34.3 | 6.7 |
| | | 3 | 8 | 34.1 | 5.4 | 8 | 37.0 | 5.3 |
| | Inner temporal | 1 | 18 | 45.4 | 4.5 | 17 | 44.7 | 6.0 |
| | | 2 | 17 | 43.8 | 5.7 | 16 | 42.8 | 7.0 |
| | | 3 | 8 | 43.3 | 6.3 | 8 | 43.5 | 3.5 |
| | Central | 1 | 18 | 19.0 | 5.4 | 17 | 18.1 | 5.3 |
| | | 2 | 17 | 18.5 | 4.5 | 16 | 18.8 | 5.5 |
| | | 3 | 8 | 17.1 | 3.0 | 8 | 17.3 | 2.7 |
